# The Still Underestimated Problem of Fungal Diseases Worldwide

**DOI:** 10.3389/fmicb.2019.00214

**Published:** 2019-02-12

**Authors:** Fausto Almeida, Marcio L. Rodrigues, Carolina Coelho

**Affiliations:** ^1^Department of Biochemistry and Immunology, Ribeirão Preto Medical School, University of São Paulo, Ribeirão Preto, Brazil; ^2^Instituto Carlos Chagas, Fundação Oswaldo Cruz (Fiocruz), Curitiba, Brazil; ^3^Instituto de Microbiologia, Universidade Federal do Rio de Janeiro, Rio de Janeiro, Brazil; ^4^Department of Molecular Microbiology and Immunology, Johns Hopkins Bloomberg School of Public Health, Baltimore, MD, United States; ^5^Department of Biosciences, College of Life and Environmental Sciences, University of Exeter, Exeter, United Kingdom

**Keywords:** fungal diseases, fungal infection, public health, fungal pathogen, fungi

## Abstract

In the past few years, fungal diseases caused estimated over 1.6 million deaths annually and over one billion people suffer from severe fungal diseases (Brown et al., 2012; Anonymous, 2017b). Public health surveillance of fungal diseases is generally not compulsory, suggesting that most estimates are conservative (Casadevall, 2017; Anonymous, 2017a). Fungal disease can also damage plants and crops, causing major losses in agricultural activities and food production (Savary et al., 2012). Animal pathogenic fungi are threatening bats, amphibians and reptiles with extinction (Casadevall, 2017). It is estimated that fungi are the highest threat for animal-host and plant-host species, representing the major cause (approximately 65%) of pathogen-driven host loss (Fisher et al., 2012). In this complex scenario, it is now clear that the global warming and accompanying climate changes have resulted in increased incidence of many fungal diseases (Garcia-Solache and Casadevall, 2010). On the basis of all these factors, concerns on the occurrence of a pandemic of fungal origin in a near future have been raised (Casadevall, 2017). In this context, to stop forgetting and underestimating fungal diseases is mandatory.

## The Burden of Human Fungal Infections

Currently, there are few antifungal drugs approved for use in humans (Pianalto and Alspaugh, 2016). The last antifungals approved for human use were characterized in 2002. This scenario may be linked to an imbalance between funding and mortality rates in the field of fungal diseases (Rodrigues, 2016). For instance, cryptococcal meningitis results in approximately 180 000 deaths annually globally (Rajasingham et al., 2017), as compared to 429 000 deaths caused by malaria or 1.6 million tuberculosis-deaths (as estimated by WHO in 2015 and 2017, respectively). However, cryptococcosis received less than 0.5% of the global research and development funding, while malaria and tuberculosis received together 35.5% of the total investment in research and development in infectious diseases (Moran et al., 2015). Current therapeutic to treat fungal diseases remain unsatisfactory, and to develop novel therapeutic alternatives a significant investment in dedicated research is required (Coelho and Casadevall, 2016). Novel antifungal are in different clinical development stages, and these new compounds such as AR-12 (Baxter et al., 2011), BHBM (Mor et al., 2015), CD101 (Zhao et al., 2016), E1210/11 (Pfaller et al., 2011), F901318 (Wiederhold et al., 2017), Ilicicolin H (Singh et al., 2012), Nikkomycin Z (Hector et al., 1990), Sampangine (Agarwal et al., 2008), Scy-078 (Lepak et al., 2015), Sertraline (Zhai et al., 2012), T-2307 (Mitsuyama et al., 2008), Tamoxifen (Dolan et al., 2009), and VT-1129 (Douglas et al., 1994). Some of these (VT-119 and Nikkomycin Z, for example) belong to new classes of drugs, i.e., they act through different mechanisms and molecular targets than the already approved drugs, and may provide a much-needed extension to the limited therapies currently available. Programs specifically offering funding to mycological research are necessary to potentiate further development of effective antifungal therapy and ultimately reduce mortality from fungal diseases.

## The Impact of Fungal Infections on Food Crops and Disproportionate Impact in Developing Countries

Fungal diseases in developing countries demand special attention. Many of the fungal diseases in Latin America are often overlooked, although they clearly fulfill the criteria to be classified as neglected human diseases (Rodrigues, 2016, 2018). The general impact of fungal pathogens on human health goes beyond the ability of fungi to infect humans, since they destroy a third of all food crops annually (Fisher et al., 2012), causing economical loss and impacting global poverty. Statistics from the 2009–2010 world harvest (www.fao.org or FAOSTAT1) suggest fungi-induced losses in five of the most important crops globally (rice, wheat, maize, potatoes, and soybean). If those losses were mitigated, these crops would have been enough to feed 8.5% of the seven billion populations in 2011 (Fisher et al., 2012). Furthermore, in a hypothetical event where these five crops were affected simultaneously, approximately 61% of the world’s population would not have food (Fisher et al., 2012). The most economically devastating fungi are *Magnaporthe oryzae*, affecting rice and wheat, followed by *Botrytis cinerea*, which has a broad host range and *Puccinia* spp., affecting wheat (Dean et al., 2012). Several high-value crops produced in the tropics, such as bananas, coffee, cacao, spices, mangos, and several nuts, are currently affected by fungal infections and these crops are not produced colder climates (Drenth and Guest, 2016). Therefore dependence of crop produced in tropical regions aggravated with a lack of biodefense and preparedness might result in disastrous economical consequences worldwide.

Fungal infections of invertebrate hosts may also impact agricultural crises due to ecological imbalance. For instance, bee broods are susceptible to fungal infections caused by genera of *Ascosphaera* and *Aspergillus* (Jensen et al., 2013), and the agricultural production worldwide is highly dependent on pollination mediated by bees (Aizen et al., 2009; Stein et al., 2017). Fungal infection of bees may precipitate a disaster (Bromenshenk et al., 2010), with unpreceded impact on agriculture and various plant species.

## The Impact of Fungal Infections on Animal Species

When fungi cause animal disease the outcomes are quite dramatic and scientists have been trying to raise attention to the effects of fungal infections in decreasing biodiversity, an effect aggravated (if not triggered) by global warming (Fisher et al., 2012; Seyedmousavi et al., 2018). Bat decimation caused by *Geomyces destructans* and several frog species by *Batrachochytrium dendrobatidis* are well known events. In invertebrates we already mentioned the case of bees, and further species may be affected but further research is needed. For example, there is a debate regarding if a marine *Aspergillus* spp. is associated with the decline of coral reefs (Kim and Harvell, 2004; Soler-Hurtado et al., 2016). Overall, the examples of species decimation in mammals, plants, and bees by fungal pathogens should prompt investigation of similar phenomenon in a wider scale, for example, other insects and in aquatic life.

## Critical Interactions of Fungi With the Environment

It is important to highlight that most of the plant-fungal interactions are beneficial to both plants and fungi, with this symbiotic interaction improving plant growth, development, foraging, acquisition of soil resources, and tolerance to stress (Zeilinger et al., 2016). For instance, *Trichoderma* induces biofertilization of crops. The addition of *Trichoderma hamatum* or *Trichoderma koningii* to the production fields can increase crop productivity up to 300% (Benitez et al., 2004). *Trichoderma* spp. also acts in the mycoparasitism, the direct attack of one fungal species on another (Harman et al., 2004; Almeida et al., 2007), being described as potential biological control agent against important phytopathogenic fungi such as *Fusarium* spp., *Rhizoctonia solani*, *Sclerotium rolfsii*, *Sclerotinia sclerotiorum*, *B. cinerea*, and *Pythium* spp. (Zeilinger and Omann, 2007). Thus, mycoparasitism-based strategies could decrease the use of agrochemicals and antifungals in crop cultivation. In this sense, a better understanding of the fungal diversity and its impact on plant-fungal interactions would be highly beneficial to improve phytopathogen control.

## The Urgency of Combatting Fungal Infections

The reduced number of antifungals impact human health not only due to lack of therapy to cure human patients. Medically approved antifungal drugs have been used for agricultural purposes for decades (Azevedo et al., 2015). Most human pathogens have environmental niches, implying that the agricultural use of medically-approved drugs imposes the concrete risk of fostering drug resistance (Verweij et al., 2009; Zavrel and White, 2015). Emergence of antifungal resistance can endanger the already limited treatments options, with calamitous effects for treatment outcomes (Perlin et al., 2017). The development of new antifungal drugs is urgent to improve both human health and agricultural production.

The worsening of global warming opens a Pandora box for fungal diseases. Some thermally intolerant fungi with current pathogenic potential should acquire the ability to survive at mammalian temperatures (Garcia-Solache and Casadevall, 2010). This threat is heightened since some fungi can take advantage of a natural selection-adaptation strategy, and consequently to adapt to higher temperature by thermal selection (de Crecy et al., 2009; Panackal, 2011).

The collaboration between all these affected disciplines would be critical in facilitating at detecting epidemics early on and preventing further spread. The concept of One Health (King et al., 2008), with integration and communication between medical doctors, veterinarians, and food safety officials has been implemented with remarkable success and should become a standard for transmissible diseases (Ghosh et al., 2018). We have included a table summarizing basic information regarding distribution and etiological agent of important human mycoses (Table 1).

**Table 1:** Examples of important human mycoses.

Fungal diseases	Distribution and etiological agent	Reference
Aspergillosis	Worldwide mycosis, caused by *Aspergillus* spp. Wide spectrum of infections in humans, but mainly in immunocompromised individuals. There are more than 250 species of *Aspergillus*, of these fewer than 40 are reported to cause infections in humans. *Aspergillus fumigatus* and *Aspergillus flavus* are the most common human pathogens.	Latge, 1999; Sugui et al., 2014
Blastomycosis	Endemic mycosis located to the south central and north central United States, caused by *Blastomyces dermatitidis* and *Blastomyces gilchristii* that can affects immunocompetent and immunocompromised individuals.	Castillo et al., 2016; McBride et al., 2017
Candidiasis	Worldwide mycosis, caused by *Candida* spp, a major cause of morbidity and mortality worldwide, and the main causative agent in systemic fungal infections. Among the *Candida* species, *Candida albicans* is the most common to cause infections in humans.	Pfaller and Diekema, 2007; Yapar, 2014
Coccidioidomycosis	Endemic to Southwestern United States and Central and South America, caused by *Coccidioides immitis* and *Coccidioides posadasii*.	Twarog and Thompson, 2015; Stockamp and Thompson, 2016
Cryptococcosis	Wordlwide distribution, caused by *Cryptococcus neoformans.* Affects immunocompromised hosts, and is a major cause of HIV-related deaths. One noticeable outbreak in Vancouver Islands of *C. gattii* in immunocompetent patients.	Casadevall and Perfect, 1998; Heitman et al., 2011; Almeida et al., 2015
Dermatophytosis	Worldwide, *Trichophyton rubrum* and *Trychophyton interdigitale* have been described as the most common to cause dermatophytosis, but many other species contribute. The most frequent type of superficial mycosis in humans, attacking skin and nails.	Weitzman and Summerbell, 1995; Bitencourt et al., 2018; Persinoti et al., 2018
Histoplasmosis	Worldwide?, *Histoplasma capsulatum* is the etiologic agent, mostly immunocompromised individuals, it is one of the most common invasive fungal pulmonary diseases.	Cano and Hajjeh, 2001; Guimaraes et al., 2006
Paracoccidiodomycosis	Endemic to Latin America, caused by thermodimorphic fungi of the *Paracoccidioides* species, is the most prevalent systemic mycosis in Latin America.	Colombo et al., 2011; Theodoro et al., 2012
*Pneumocystis* pneumonia	Worldwide, caused by *Pneumocystis jiroveci*, affects patients that were immunosuppressed, such as cancer patients receiving chemotherapy or HIV patients.	Thomas and Limper, 2004; Morris and Norris, 2012
Sporotrichosis	Worldwide, subcutaneous and subacute/chronic disease caused by dimorphic fungus of the genus *Sporothrix* that affects humans and other mammals.	Chakrabarti et al., 2015; Conceicao-Silva and Morgado, 2018


The negative impact of any infectious disease is usually higher in neglected populations. Lack of access to healthcare, underfunded healthcare, and delayed diagnosis lead to higher burden of fungal diseases. The economy of countries in developing countries strongly relies on agricultural production. Another fungal blight, if not managed properly, can still have disastrous economical and societal consequences.

The old adage “know your enemy” illustrates the major point of this essay: significant investment in fungal research, including fungal biology, human and plant pathogenicity, therapeutic agents, diagnostic tools, and vaccines, has the potential to avoid a global catastrophe. This call has been made by most, if not all, prominent fungal researchers (Molloy et al., 2017; Casadevall, 2018). We urge more attention to fungal biology and pathogenesis globally with special attention to diseases affecting developing countries, as a safeguard for prevention of losses in agriculture, environmental losses, and damage to human health.

## Author Contributions

All authors listed have made a substantial, direct and intellectual contribution to the work, and approved it for publication.

## Conflict of Interest Statement

The authors declare that the research was conducted in the absence of any commercial or financial relationships that could be construed as a potential conflict of interest.
